# Updated total IgE reference intervals in Norwegian adults

**DOI:** 10.1002/iid3.751

**Published:** 2022-12-15

**Authors:** Erik Wilhelm Vinnes, Birthe Skarbø, Tore Wentzel‐Larsen, Marit S. Sylte, Torunn Oveland Apelseth

**Affiliations:** ^1^ Department of Medical Biochemistry and Pharmacology Haukeland University Hospital Bergen Norway; ^2^ Centre for Clinical Research, Haukeland University Hospital Bergen Norway; ^3^ Centre for Child and Adolescent Mental Health, Region East & South Oslo Norway; ^4^ Norwegian Centre for Violence and Traumatic Stress Studies Oslo Norway; ^5^ Department of Immunology and Transfusion Medicine Haukeland University Hospital Bergen Norway; ^6^ Norwegian Armed Forces Medical Services Sessvollmoen Norway; ^7^ Department of Clinical Science University of Bergen Bergen Norway

**Keywords:** immunoglobulin E, in vitro allergy testing, reference intervals, reference range, reference values, total IgE

## Abstract

**Background:**

It is important and expected of laboratories to provide updated reference intervals to the clinician. As no recent publications report adult total IgE reference intervals on a Scandinavian population, the aim of our study was therefore to provide an estimate on healthy Norweigian adults.

**Methods:**

A reference interval study was conducted in accordance to CLSI guidelines. Samples were collected from *n* = 252 presumably healthy adult participants enrolled through the regional blood donation program. Total IgE measurements were performed on the ImmunoCAP^TM^ platform (Thermo Fisher Diagnostics) traceable to the WHO‐reference standard (75/502) for total IgE measurements.

**Results:**

An upper 95% total IgE reference limit was estimated to 302 kU/L (90% CI 177–388 kU/L), and the 97.5% percentile was estimated to 391 kU/L (90% CI 344–560 kU/L). No significant differences were found between participants who self‐reported having an allergic disease and participants who did not self‐report having an allergic disease.

**Conclusion:**

Our results and other recent publications find markedly higher values than adult reference intervals established four decades ago which still remain widely used by clinical laboratories. We therefore recommend total IgE reference intervals should be critically reviewed and updated.

## Graphical abstract

We performed a reference interval study of total IgE on presumably healthy adult participants enrolled through the regional blood donation program. Analysis was performed using the ImmunoCAP^TM^ platform. In summary we found markedly higher values than adult reference intervals established four decades ago which still remain widely used by clinical laboratories. We therefore recommend total IgE reference intervals should be critically reviewed and updated.

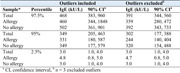

## 1 INTRODUCTION

The measurement of immunoglobulin E (IgE) is an important test of sensitization used for the evaluation of allergic diseases. It is however widely known that serum *total‐IgE* measurements by itself serves a limited role in the diagnosis of allergic disease.^1^ High values may be seen in a multitude of non‐atopic conditions including primary immunodeficiencies, neoplastic disease, parasitic and inflammatory diseases. However, the significance of low values remains uncertain and needs to be further explored in the clinical setting.^2^

It is important for clinical laboratories to provide updated reference intervals in accordance to conventions^3^, ^4^ and currently no publications provide updated adult total IgE reference intervals on a Scandinavian population measured on modern immunoassays. Reference values have been investigated on a large European population (*n* = 6670) by Carosso et al.^5^ in which both Norwegian and Swedish participants were included. However nationality‐specific reference intervals were not provided in the publication. Norwegian clinical laboratories have therefore largely opted to rely on manufacturer provided reference intervals^6^ referring to values established more than four decades ago by Zetterstrøm et al.^7^ The aim of our study was therefore to establish an updated reference interval in healthy Norwegian reference subjects.

## 2 METHODS AND MATERIALS

### 2.1 Study population and ethics

Blood samples were collected from 245 presumably healthy blood donors participating in the regional routine blood donation program at Haukeland University Hospital, Bergen, Norway. A further of seven participants were recruited amongst laboratory personnel in the age groups >60 years of age, for a total of 252 participants.

The general exclusion criteria for the blood donation program require that the blood donor could not previously have experienced any anaphylactic episode, but donors may have an allergic disease managed by per oral antihistamines or topical treatment. Participants in our study were required to answer a questionnaire inquiring whether they had experienced *any previous allergic reaction and/or suspects they may have an allergic disease*. In the case of an affirmative answer to at least one of the above, the participant was defined as having *self‐reported having an allergic disease*.

Reference intervals were calculated on *all* participants and the two partitioned subgroups who *self‐reported having an allergic disease* and participants who *did not report having an allergic disease*. The study was approved by the Regional Ethics Committee (2017/1179/REK Vest). All study participants provided written informed consent.

### 2.2 Sample handling and laboratory methods

The study was performed during October 2017–January 2018 at the Department of Medical Biochemistry and Pharmacology, Haukeland University Hospital. Blood samples were collected by venipuncture using 3.5 ml serum‐separation gel tubes (Becton Dickinson Vacutainer), centrifuged at 3000*g* for 10 min and stored at 4°C before analysis of total IgE was performed within a maximum of 2 working days. Serum samples were analyzed on a daily basis on an ImmunoCAP^TM^ 1000 instrument (Thermo Fisher Diagnostics) as a part of the department's clinical routine analysis. The ImmunoCAP^TM^ immunoassay for total IgE is traceable to the WHO‐reference standard for total IgE measurements, standard 75/502.^6^

Internal quality controls (analytical CV 7.9%) were performed on a daily basis, and measurements were monitored by two separate external quality assurance (EQA) programs, *DEKS HK18 Immunoglobulin E* and *Quality Club* respectively. Our total‐IgE measurements were in good agreement with other participants in both EQA programs in regard to the *trueness* of the measurements.

### 2.3 Statistical analysis

Reference limits were estimated by nonparametric analysis as 2.5%, 95%, and 97.5% percentiles including 90% confidence intervals (CI). CIs were computed by an interpolation procedure.^8^ Computations for 97.5% and 95% percentiles were performed for log transformed IgE and estimates were transformed back. Outlier sensitivity analysis was performed by Tukey's method (on log transformed IgE) as suggested by CLSI guidelines, declaring as outliers observations more than 1.5 times the interquartile range above the third quantile, or more than 1.5 times the interquartile range below the first quantile.^4^ Analyses were performed in R (version 4.1.2, The R Foundation). Confidence intervals for percentiles, with interpolation, were computed using the function “quantile_confint_nyblom” in in the R package “quantileCI” made available by M. Höhle at a GitHub repository, (https://github.com/hoehleatsu/quantileCI, accessed 28.04.2022).

## 3 RESULTS

In total 252 participants were recruited, however 3 participants were excluded due to a lack of completing the participant's questionnaire and a further 4 participants were excluded due to sampling handling error on behalf of the laboratory. Age and sex characteristics of the included 245 participants are presented in Table 1.

**Table 1:** Characteristics of study participants

	All participants	Participants self‐reported having an allergic disease	Participants *not* self‐reported having an allergic disease
Age (years)	*N* = 245	*N* = 112	*N* = 133
19–29	62	30	32
30–39	41	23	18
40–49	43	22	21
50–59	40	15	25
60–69	39	20	19
≥70	20	2	18
Sex			
Female	124	60	64
Male	121	52	69

Of the 245 included participants, 112 individuals self‐reported *having an allergic disease* and/or *previously having experienced an allergic reaction*. Reference values were estimated on all participants (*n* = 245) and on the two subgroups, the first consisting of individuals who self‐reported having an allergic disease (*n* = 112), and the second group who did not self‐report having an allergic disease (*n* = 133).

**Figure 1:**
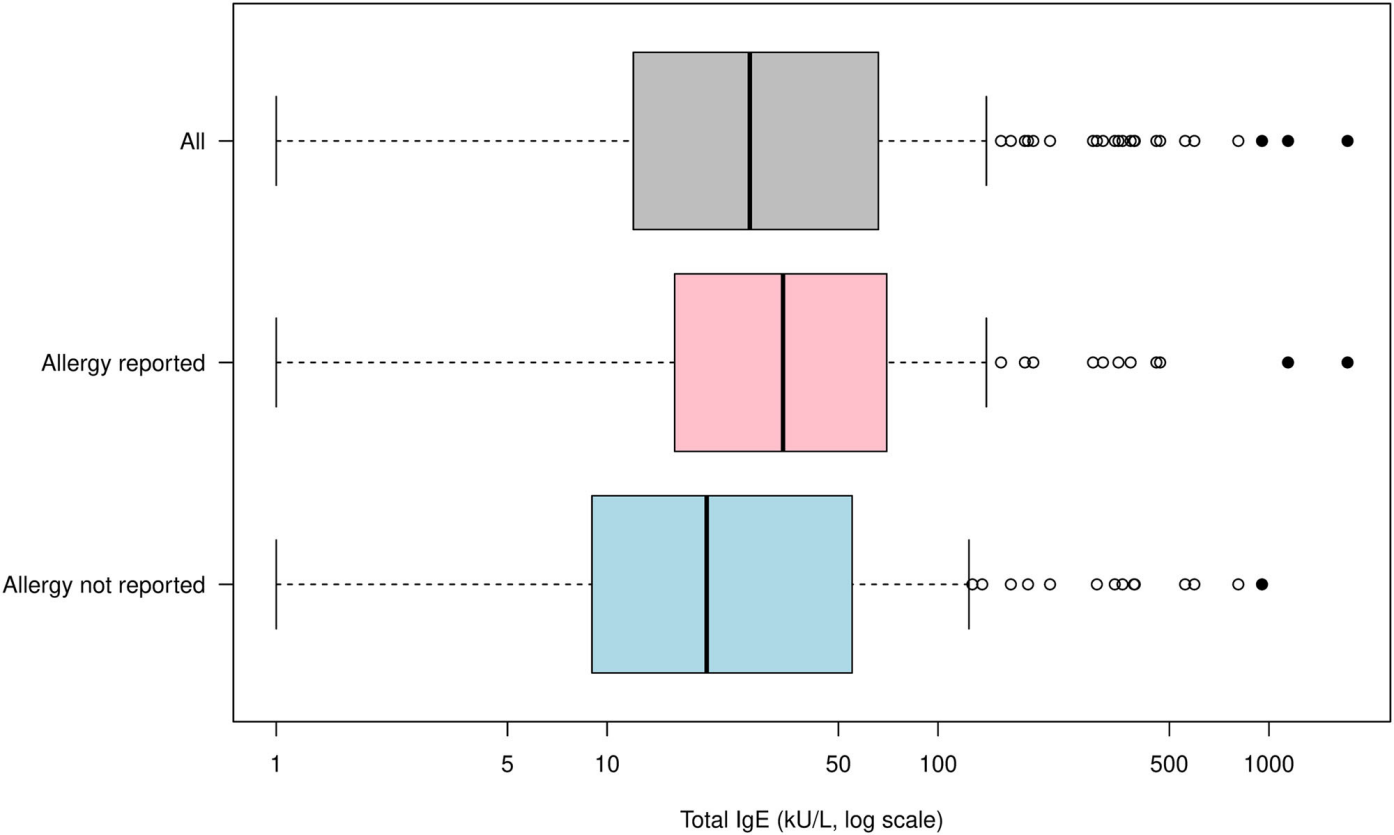
Total IgE values (kU/L) amongst study participants. Grey displays *all* participants (*n* = 245). Pink displays the sub‐group who *self‐reported having allergy* (*n* = 112) and blue shows participants who *did not self‐report having allergy* (*n* = 133). Data points evaluated as outliers are shown as solid points (*n* = 3 excluded outliers).

Total IgE results are shown in Figure 1 and reference values (percentiles) for IgE with 90% CIs are shown in Table 2. Three IgE observations were identified as outliers, all three in the upper range between 950 and 1730 kU/L. Exclusion of outliers generally gave somewhat lower percentiles, particularly for the 97.5 percentiles, and did almost not change the 2.5 percentiles.

**Table 2:** Reference values shown as 2.5%, 95%, and 97.5% percentiles with their 90% confidence intervals

		Outliers included		Outliers excluded^b^	
Sample^a^	Percentile	IgE (kU/L)	90% CI	IgE (kU/L)	90% CI
**Total**	97.5%	468	383, 960	391	344, 560
Allergy		460	344, 1848	359	280, 472
No allergy		502	361, 901	392	343, 733
**Total**	95%	349	205, 463	302	177, 388
Allergy		331	180, 587	244	140, 404
No allergy		349	177, 579	320	154, 488
**Total**	2.5%	3.0	1.0, 4.0	3.0	1.0, 4.0
Allergy		4.8	0.8, 5.0	4.7	0.8, 5.0
No allergy		3.0	1.0, 4.0	3.0	1.0, 4.0

Abbreviations: CI, confidence interval; IgE, Immunoglobulin E.

^a^ Allergy and no allergy refers to participants who self‐reported having and who did not self‐report having allergy.

^b^ *n* = 3 excluded outliers.

## 4 DISCUSSION

### 4.1 Strengths and weaknesses

A strength of our study is the estimation of reference values by direct method on presumably healthy participants in accordance to CLSI guidelines.^3^, ^4^ It is also a strength that measurements were performed on modern methods and instrumentation currently widely used in clinical laboratories and traceable to the WHO reference material.^9^ Our laboratory is certified in accordance to ISO15189:2012 and is regularly audited by the Norwegian accreditation body. Furthermore our total‐IgE levels are in accordance with other clinical laboratories evaluated by two independent external quality assessment schemes. We had enough participants to perform non‐parametric estimations and were also able to provide calculations on two partitioned subgroups, participants *self‐reported allergy* and participants who *did not self‐report allergy*, respectively.

It would have been preferable to have included more study participants as this would have conferred more precise confidence intervals for the estimation of the 95% and 97.5% percentiles. Although even with wide confidence intervals, our estimates may be in accordance with, though somewhat higher than other recently published studies on European and North American populations.^5^, ^10^, ^11^ Our data seems to be more in line with total IgE reference values found on Middle Eastern populations.^12^, ^13^ Thus our data suggests the manufacturer provided reference values should be updated as they present substantially lower values (Thermo Fisher diagnostics, upper adult reference value of 114 kU/L^6^) compared to more recent literature reporting reference intervals in the order of 148–600 kU/L.^5^, ^11^, ^12^, ^13^, ^14^

Our study exhibits the weakness of not recording information regarding smoking habits amongst the participants, therefore we are not able to provide partitioned estimates differentiating between participants who were smokers and non‐smokers. It has been widely shown that smokers exhibit higher total IgE levels compared to nonsmokers,^5^, ^15^ thus this would have been a useful parameter in our study. On the other hand, updated data from the Norwegian Institute of Public Health records shows a clear and concurrent historical decline in smoking habits in the Norwegian population. According to data from 2017, only approximately 10% of the population reported smoking sporadically or on a daily basis.^16^

In our study a large proportion of the participants self‐reported having an *allergic disease*, *n* = 112 of 245 participants (46%). Food (17%), unspecified pollen (16%), feline (15%), house dust mite (13%), grass/timothy (12%) and birch (9%) allergies were the most common *self‐reported allergies* in our investigation. A meta‐analysis by Nwuro et al.^18^ found a pooled *lifetime* and *point* prevalence of self‐reported food allergy of 17.3% and 5.9%, respectively in European studies performed from 2000 to 2012. In our study, the prevalence of self‐reported food allergies may thus be in correspondence to what has been reported in the literature.^18^

A possible explanation for the high prevalence amongst our participants might be the *loose* formulation of the questionnaire being: *Have you ever had an allergic reaction or do you suspect having an allergy?* We did not explore the high incidence of participants self‐reporting having an allergic disease in further detail as we considered this to be beyond the scope of our reference interval investigation. As is shown in Table 2, there were no significant differences in the upper 95% and 97.5% reference values on participants *self‐reporting having an allergic disease* (*n* = 112) versus *participants not reporting having an allergic disease* (*n* = 133).

### 4.2 Comparison of our total IgE values with recently published reference values

Our upper limits may be in concordance with, although somewhat higher than findings by Martins et al.^11^ on a Salt Lake City population (upper 97.5% limit of 214 kU/L on *n* = 128 adults), and Carrosso and colleagues (95th percentiles of 169 kU/L in males and 148 kU/L in females) on a large European population. In the same study, the 99th percentile of kU/L was 341 and 300 kU/L in males and females, respectively).^5^ Our upper limits are also higher than the upper limit (97.5th percentile) of 297 kU/L amongst *n* = 226 males and 257 kU/L among *n* = 331 females reported by Simoni et al.^10^ on an Northern Italian population. On the other hand our upper 97.5% limit is considerably lower than the 97.5% limit of 602.5 kU/L reported on a (*n* = 546) Kuwaiti population, and may be in line with the 95% limit of 250 kU/L on an Iranian population.^13^

Our reference intervals were established as a single center study. Each laboratory must however evaluate if these results are applicable to their respective populations.^3^, ^4^, ^18^ Ideally future total IgE‐reference interval studies should strive to include larger sample sizes in addition to including multiple centers. In general it is important to be cognisant when comparing reference intervals and be aware whether one is presenting a two‐sided interval (2.5%–97.5%) or an one‐sided interval (<95%, i.e., 0%–95%). Clinical laboratories must take this into consideration when transferring a reference interval from the literature or the manufactuer's package insert, and should state clearly towards the clinicians what percentiles are being presented. In regard to total IgE‐levels, the literature on a whole and current practice by laboratories are inconsistent in regard to choosing a two‐sided or one‐sided reference interval as both are being widely used concurrently. It is clear that this confers a large difference in regard to what the laboratory will flag as being “pathological.” We thus recommend this should be discussed in further detail by clinical allergists in tandem with laboratorians.

### 4.3 Conclusion

In conclusion, we have estimated adult total IgE reference intervals on presumably healthy subjects. We note however that our upper reference limits, in addition to findings in the recent literature^5^, ^10^, ^11^, ^12^, ^13^ are markedly higher than the upper limit of 114 kU/L currently used by many European clinical laboratories.^6^ We therefore recommend that adult total IgE reference intervals should be critically reviewed and updated in accordance to more recent publications.

## AUTHOR CONTRIBUTIONS

Erik Wilhelm Vinnes planned the study, wrote the manuscript, and performed search of the literature. Birthe Skarbø organised sampling of the study participants and performed laboratory analysis. Tore Wentzel‐Larsen performed statistical analysis and contributed to the writing of the manuscript. Marit S. Sylte and Torunn Oveland Apelseth provided guidance and supervision as senior scientists and contributed to the writing of the manuscript. All authors have provided revisions to the manuscript and reviewed the final draft.

## CONFLICT OF INTEREST

The authors declare no conflict of interest.

## Data Availability

Data may be available upon reasonable request to the corresponding author. Restrictions may apply due to ethical considerations or third party restrictions.
